# Psychosocial determinants of cardiovascular events among black Americans with chronic kidney disease or associated risk factors in the Jackson heart study

**DOI:** 10.1186/s12882-021-02594-6

**Published:** 2021-11-11

**Authors:** Nrupen A. Bhavsar, Clemontina A. Davenport, Lexie Zidanyue Yang, Sarah Peskoe, Julia J. Scialla, Rasheeda K. Hall, Crystal C. Tyson, Tara Strigo, Mario Sims, Jane Pendergast, Lesley H. Curtis, L. Ebony Boulware, Clarissa J. Diamantidis

**Affiliations:** 1 Division of General Internal Medicine, Duke University School of Medicine, 200 Morris St, 3rd Floor, NC 27701 Durham, USA; 2 Department of Biostatistics and Bioinformatics, Duke University School of Medicine, Durham, NC USA; 3 Division of Nephrology, Duke University School of Medicine, Durham, NC USA; 4 Departments of Medicine and Public Health Sciences, University of Virginia School of Medicine, Charlottesville, VA USA; 5 University of Mississippi School of Medicine, Jackson, MS USA; 6 Department of Population Health Sciences, Duke University School of Medicine, Durham, NC USA

## Abstract

**Background:**

Individuals with chronic kidney disease (CKD), hypertension (HTN), or diabetes mellitus (DM) are at increased risk for cardiovascular disease (CVD). The extent to which psychosocial factors are associated with increased CVD risk within these individuals is unclear. Black individuals experience a high degree of psychosocial stressors due to socioeconomic factors, environment, racism, and discrimination. We examined the association between psychosocial factors and risk of CVD events among Black men and women with CKD and CKD risk factors in the Jackson Heart Study.

**Methods and Results:**

We identified 1919 participants with prevalent CKD or CKD risk factors at baseline. We used rotated principal component analysis - a form of unsupervised machine learning that may identify constructs not intuitively identified by a person - to describe five groups of psychosocial components (including negative moods, religiosity, discrimination, negative outlooks, and negative coping resources) based on a battery of questionnaires. Multiple imputation by chained equation (MICE) was used to impute missing covariate data. Cox models were used to quantify the association between psychosocial components and incident CVD, defined as a fatal coronary heart disease event, myocardial infarction, cardiac procedure (angiography or revascularization procedure), or stroke. Of the 929 participants in the analysis, 67% were female, 28% were current/former smokers with mean age of 56 years and mean BMI of 33 kg/m^2^. Over a median follow-up of 8 years, 6% had an incident CVD event. In multivariable models, each standard deviation (SD) increase in the religiosity component was associated with an increased hazard for CVD event (hazard ratio [HR] = 1.52, 95% CI: 1.09–2.13).

**Conclusions:**

Religiosity was associated with CVD among participants with prevalent CKD or CKD risk factors. Studies to better understand the mechanisms of this relationship are needed.

**Supplementary Information:**

The online version contains supplementary material available at 10.1186/s12882-021-02594-6.

## Introduction

Over 30 million adults in the United States have chronic kidney disease (CKD) [1]. Individuals with CKD experience increased risk for cardiovascular disease (CVD) and are more likely to die of CVD than other causes [1, 2]. This heightened CVD risk is particularly striking among Black adults with CKD, who are more likely to die of CVD than white adults [3]. Several studies examining the association between psychosocial variables and CVD events among the general public suggest a relation between greater levels of anxiety, anger, discrimination, hostility, and stress and adverse CVD outcomes [4, 5]. Black individuals experience a high degree of psychosocial stressors related to socioeconomic factors, environment, racism, and discrimination [6–8]. To our knowledge, no studies have evaluated how these stressors may increase risk for CVD among Black individuals with CKD or major CKD risk factors.

Psychosocial variables that may negatively impact health include religiosity, negative moods (anger and hostility), discrimination, poor coping resources (low social support and social status), and negative outlooks (John Henryism and pessimism). Previous studies have shown that these factors may have negative cardiovascular consequences, including increased risk for incident and recurrent heart disease [9–11], and they have been conceptualized to impact physiological health through multiple mechanisms, including heightened stress response and an increase in unhealthy behaviors [12]. High blood pressure and uncontrolled blood glucose are two major risk factors for CKD. Heightened psychosocial stress can further elevate blood pressure and stimulate cortisol secretion which, among other effects, raises blood glucose. Coupled with stress induced cigarette and alcohol use for coping, these effects could exacerbate CVD in adults with CKD and CKD risk factors [13, 14]. However, much of the research to date has examined the relationship of these factors with intermediate health outcomes, such as blood pressure, and have lacked associations to hard clinical events such as myocardial infarction or stroke [15]. Moreover, few studies have examined this relation among Black adults. Studies elucidating the association between psychosocial factors and CVD risk, particularly among Black adults with CKD or CKD risk factors, could identify important targets for addressing increased risks of CVD in this population.

We examined the association between psychosocial factors and incident CVD events among Black men and women with CKD or CKD risk factors in the Jackson Heart Study (JHS). We hypothesized that a greater burden of negative psychosocial factors would increase the risk for CVD events among JHS participants.

## Methods

### Study participants

The JHS is a National Heart, Lung, and Blood Institute (NHLBI) sponsored single-site prospective cohort study examining the causes of CVD in non-institutionalized adult Black residents of the tri-county area (Hinds, Madison, and Rankin) of the Jackson, Mississippi, metropolitan area. The study design has been previously described [16]. The baseline cohort includes 5301 men and women aged 21–94 years recruited from 2000 to 2004. Baseline data were collected using a self-administered questionnaire, in-home interview, and in-clinic study visits. The study was approved by the institutional review boards of the University of Mississippi Medical Center, Jackson State University, and Tougaloo College, and all participants provided written informed consent.

To identify characteristics relevant to individuals with or at risk for CKD, we restricted our study sample to JHS participants with baseline CKD or major CKD risk factors defined as prevalent hypertension and/or diabetes mellitus at baseline. CKD was defined as an estimated glomerular filtration rate (eGFR) < 60 mL/min/1.73 m^2^ using the CKD-EPI formula or an albumin-to-creatinine ratio of ≥30 mg/g on spot urine collection or 24-h urine collection if the former was unavailable [17]. Hypertension was determined using the JNC-8 criteria for detection of hypertension (i.e., self-reported use of hypertension medication or age ≥ 60 yrs.: SBP ≥150 or DBP ≥90 mmHg [average of 2 blood pressure measurements]; age < 60 yrs.: SBP ≥ 140 or DBP ≥ 90 mmHg; or age ≥ 18 yrs. with CKD or diabetes: SBP ≥ 140 or DBP ≥90). Diabetes was defined based on the American Diabetes Association 2010 guideline; participants with (1) fasting glucose ≥126 mg/dL or, (2) hemoglobin A1C ≥ 6.5% or, (3) use of diabetic medication confirmed by inspecting pill bottles within 2 weeks prior to the clinic visit were classified as having diabetes. Participants with a history of CVD and those without baseline hypertension, diabetes, and CKD (i.e., participants without all three conditions) were excluded.

### Independent variables: psychosocial components

A total of 15 psychosocial variables were measured at baseline: daily discrimination, lifetime discrimination, burden of lifetime discrimination, global stress, John Henryism, spirituality, organized religion, non-organized religion, religious coping, hostility, anger in, anger out, pessimism, social support, and perceived social status.

Perceived discrimination was assessed with instruments validated for use in the JHS cohort [18]. The dimensions of perceived discrimination included daily discrimination, lifetime discrimination, and burden of lifetime discrimination. For daily discrimination, participants rated the frequency of occurrence of 9 discriminatory experiences (e.g., poor service at a restaurant) on a scale from 0 to 7 (none to several times per day) in response to the question “How often on a day-to-day basis do you have the following experiences?” The daily discrimination score comprised the mean score across these 9 experiences. Lifetime discrimination, which measures unfair treatment in 9 social domains (e.g., school, housing), was calculated by summing binary counts (no = 0, yes = 1) from these domains to generate a total score (range 0–9). Burden of lifetime discrimination was quantified through a Likert scale of 1) how stressful each unfair treatment was (“not stressful” [0] to “very stressful” [3]); 2) interference with living a full and productive life (“not at all” [0] to “a lot” [3]); and 3) whether it increased difficulty in life (“not at all” [0] to “a lot” [3]). Burden of lifetime discrimination was restricted to persons who reported at least 1 instance of lifetime discrimination. Responses were summed to generate the burden of lifetime discrimination score (range 0–9) [5].

Stress was evaluated using the Global Perceived Stress Scale (GPSS), which was adapted and validated for use within the JHS from the Survey of Recent Life Experiences scale, Perceived Stress Scale, and Life Events Scale [19]. The questionnaire captured 8 potential stress-inducing contexts, including employment, relationships, neighborhood, caring for others, legal problems, medical problems, racism and discrimination, and meeting basic needs. The severity of stress experienced for each item during the 12-month period preceding the baseline examination was rated on a Likert scale (“not stressful” [0] to “very stressful” [3]). The GPSS score was calculated by summing responses to the 8 items on the questionnaire (range 0–24).

John Henryism was measured using the 12-item John Henryism Scale for Active Coping, which evaluates the extent to which one engages in active coping in order to overcome socioeconomic stress [20]. This scale includes prompts like “I’ve always felt that I could make of my life pretty much what I wanted to make of it” and “Once I make up my mind to do something, I stay with it until the job is completely done.” Responses are scored on a Likert scale (“completely false” [0] to “completely true” [3]) and summed across the 12 items to generate a composite John Henryism score (range 0–36). Higher scores indicate greater unnecessary effort to engage in coping activities with social and economic adversity that can result in negative health consequences (i.e., poor coping).

Religiosity was measured using 4 independent subscales: spirituality, organized religion, non-organized religion, and religious coping. Spirituality was measured using the Daily Spiritual Experience Scale, a 6-item questionnaire targeting daily experiences with God and spirituality [21]. The responses range from “never” (0) to “many times a day” (5) and are summed to get a continuous measure from 0 to 30. Organized religion was assessed by the question “How often do you attend the main worship service of your church or otherwise participate in organizational religion?” with responses ranging from “not at all” (0) to “nearly every day” (5). Non-organized religion was assessed by the question “Within your religious or spiritual tradition, how often do you pray privately or meditate in places other than at church, mosque, temple, or synagogue?” with responses ranging from “never” (0) to “more than once a day” (7). Religious coping was assessed by the question “To what extent is your religion or spiritual tradition involved in understanding or dealing with stressful situations in any way?” with responses ranging from “not involved at all” (0) to “very involved” (3).

Hostility was examined using the sum of 27 true/false items derived from the Cook-Medley Hostility Scale, which included measures of cynicism, hostile affect, and aggressive response [19, 22]. An affirmative response to each item on this scale received a score of 1, and a negative response received a score of 0. A summary score was generated by calculating the sum of responses (range 0–27).

Anger was assessed using a 16-item scale, termed the Spielberger trait anger scale, which comprises 2 separate 8-item sub-scales for anger in and anger out [23]. Participants rated each questionnaire item on a Likert-type scale range from “almost never” (0) to “almost always” (3), based on how often they react to statements such as “I keep things in” or “I am secretly quite critical of others” (anger-in) and “I express my anger” or “I do things like slam doors” (anger-out). Responses were summed across items in each subscale to generate 2 anger scores, each ranging from 0 to 24.

Pessimism was assessed using the 3-item revised Life Orientation Test [24]. Participants responded to statements such as “If something can go wrong for me, it will,” using a 4-point scale ranging from “strongly disagree” (0) to “strongly agree” (3). Scores were summed from the 3 items to calculate a summary score ranging from 0 to 9.

Social support was assessed using the SOCA, which is a subset of 3 questions derived from the Lubben Scale [25]. This construct collects information on structural components of social support. Scores from Likert-type responses were summed to calculate a social support score ranging from 0 to 12. Perceived social status was assessed using a single question asking participants to rate their standing in the community on a reverse coded scale ranging in value from “high standing” (1) to “low standing” (10).

The primary independent variables were psychosocial components derived from conducting principal component analysis (PCA) on the 15 psychosocial variables measured at baseline. We conducted PCA using the R function “principal” in the psych package [26] on 929 participants with complete information on psychosocial variables to reduce the 15 psychosocial variables of interest into fewer conceptually related, though uncorrelated, linear combinations of the original 15 (i.e., components) [27]. Varimax rotation was performed to facilitate interpretability, and any components with eigenvalues ≥1 were retained. Psychosocial variables with a factor load ≥0.4 loaded favorably on a given component, and were used to represent the dimension of interest. As a comparison, we conducted PCA and all other analyses using the 716 participants with complete information on psychosocial variables and covariates.

### Covariates

Demographic and clinical information from the baseline exam included age, sex, BMI (kg/m^2^), comorbidities such as hypertension, diabetes and CKD, smoking status dichotomized as “never” or “former/current,” use of antihypertensive medications, parental history of stroke or heart disease, and dyslipidemia defined as total cholesterol ≥200, LDL ≥130, or statin use. Notably, we adjusted for both hypertension and antihypertensive medication use because the derived definition of hypertension only included *self-reported* medication use. As CVD outcomes are of interest, actual medication use was deemed important as a potential confounder. Occupation categories were classified as wage-earning and non-wage-earning. Income was defined as ≤1.5 or > 1.5 times the poverty level. The poverty level was determined based on the visit year, family size, and the number of children < 18 years of age within the household. Education level was categorized as < or ≥ high school. Nutrition level and physical activity were categorized as ideal, intermediate, and poor health. Alcohol use was dichotomized as yes and no. Depression was derived using Hassles and moods B form, with the answers converted to a scale of 0–3 and a score of ≥16 signifying depression.

### Dependent variable: CVD events

A CVD event was defined through 2015 as a definite/probable myocardial infarction, a cardiac procedure, fatal coronary heart disease event, or a definite/probable stroke. All events were adjudicated and the adjudication procedures have been described previously [16]. Briefly, outcomes were recorded during the annual telephone interview with participants and their family members and at study visits during exams 2 (2005–2008) and 3 (2009–2013) of the JHS study. During the interviews and examinations, trained staff identified interim medical events, including new health events, diagnostic tests, hospitalizations, new diagnoses, and death. These were subsequently confirmed by review of medical records, including discharge summaries, International Classification of Diseases, Ninth Revision codes, and procedure codes. Participant mortality was also identified from the Mississippi State Department of Health, review of death certificates and hospital charts, obituaries, and linkage to the National Death Index through 2015.

### Statistical analyses

We described demographic and clinical characteristics of study participants overall and compared characteristics according to their development of incident CVD using t-tests and Wilcoxon Rank Sum test for mean and median values of continuous variables, respectively, and using chi-square tests for categorical variables. We employed Cox regression models to quantify the association between the derived psychosocial components and incident CVD events, while adjusting for demographic and clinical covariates. We examined the extent and frequency of missing covariates, including diabetes, dyslipidemia, systolic blood pressure, diastolic blood pressure, antihypertensive medications, smoking status, BMI, parental history of CVD, employment, alcohol use, nutrition, and depression. While HDL is a known contributor to cardiovascular disease, it is not included in the final model to avoid overfitting and collinearity with dyslipidemia. We used multiple imputation chained equation (MICE) to create 10 imputed datasets through the R package “mice” [28] with the predictive mean matching method to impute the non-psychosocial covariates.

The association between psychosocial components and CVD was examined continuously (i.e., per standard deviation increase) and categorically (i.e., at the median and by tertile of measure) using Cox models adjusted the previously stated confounders. The proportional hazards assumption was tested for the models described and no significant violations were found (all *p* > 0.05). Analyses were then stratified by age (median cutoff) and sex, as there are age and gender differences in psychosocial measures [29, 30]. A test for interaction was conducted to determine if age and sex modified the association between the derived psychosocial components and incident CVD by adding an interaction term to the aforementioned Cox models.

In sensitivity analyses, the Cox model was fit using competing risk methods [31] to account for informative censoring due to non-CVD related death. We also fit a model that included individuals with missing CKD status (by grouping them as an additional category) to explore patterns of missing data and whether individuals with missing CKD status had different risks for the outcomes as compared to individuals without missing CKD status. PCA were conducted for all sensitivity analyses to derive psychosocial components. Additionally, the main Cox model was rerun with HDL included as a covariate. Analyses were performed using R 4.0.3 (R Core Team (2020), Vienna, Austria) and SAS 9.4 (SAS Institute, Cary, NC).

## Results

### Study sample

**Fig. 1:**
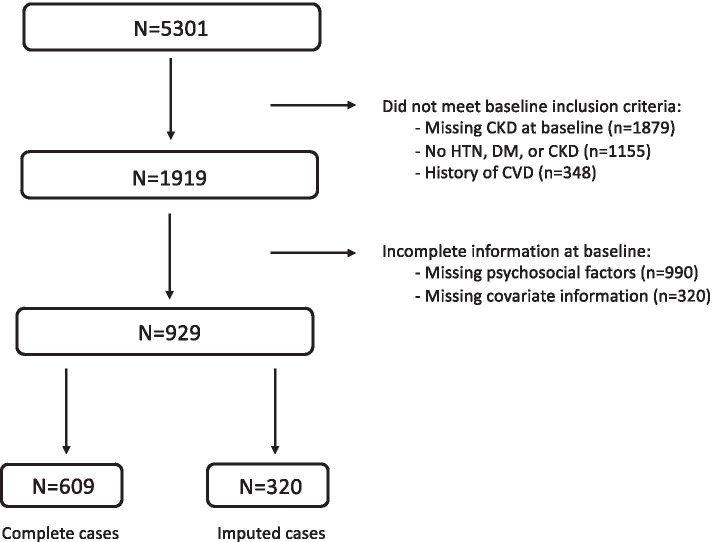
Flow chart of participants who met inclusion/exclusion criteria for the study population

Of the 5301 JHS participants, 1879 had missing CKD status at baseline; 1155 did not have hypertension, diabetes, or CKD at baseline; 348 had prevalent CVD at baseline; and 990 had missing/incomplete psychosocial information. A further 320 (34%) had missing covariate information. Of these, 78% had only 1 variable missing (Additional file 1: Appendix 1). The highest proportion of missing data occurred for income (15.9%), dyslipidemia (9.5%), nutrition (7.8%), and antihypertensive medication use (6.5%) (Additional file 1: Appendix 2). The other covariates imputed had no more than 2.3% of missing values. After imputing missing data in these 320 participants, the resulting final analysis cohort included 929 participants (Fig. 1).

The study participants were 67% female with a mean age of 56 years and a mean BMI of 33 kg/m^2^. Over 69% of the participants had at least a high school level of education, 61% were wage earning (i.e., working for pay), and 57% lived at more than 1.5 times the poverty level. At baseline, 30% of the participants had diabetes, 92% had hypertension, and 21% had CKD. Approximately 76% of the sample were using antihypertensive medications, 28% were current or former smokers, 59% had dyslipidemia, and 52% had a parental history of CVD. (Table 1).

**Table 1:** Baseline characteristics of the study population

	Overall	No CVD	Incident CVD	***p***-value
n	929	869	60	
**Age, years (mean, SD)**	56.4 (11.4)	56.0 (11.3)	61.4 (11.3)	***p*** **< 0.01**
**Female (%)**	66.6	67.3	56.7	**0.1**
**Education (%)**				**0.6**
<High school	30.8	30.5	35	
≥High school	69.2	69.5	65	
**Income (%)**				**0.1**
≤1.5x poverty level	32.2	31.5	41.7	
> 1.5x poverty level	67.8	68.5	58.3	
**Employment (%)**				**0.03**
Wage-earning	60.9	61.9	46.7	
Non-wage-earning	39.1	38.1	53.3	
**BMI (mean, SD)**	32.8 (7.2)	32.8 (7.3)	32.7 (7.0)	**0.9**
**Smoking status (%)**				
Former/current	27.8	26.7	43.3	**0.01**
**Alcohol use (%)**	41.6	41.7	40	**0.9**
**Nutrition (%)**				**0.5**
Poor	53	53.3	48.3	
Intermediate	45.9	45.5	51.7	
Ideal	1.2	1.3	0	
**Physical Activity (%)**				**0.04**
Poor	46.8	45.8	61.7	
Intermediate	33.2	34.1	20	
Ideal	20	20.1	18.3	
**Depression (%)**	46.6	46.5	48.3	**0.9**
**Diabetes status (%)**	29.8	28.5	48.3	**< 0.01**
**Hypertension status (%)**	91.9	91.7	95	**0.5**
**Antihypertensive medication use (%)**	78.9	78	91.7	**0.02**
**Chronic kidney disease (%)**	20.9	**19.**4	41.7	**< 0.01**
**Dyslipidemia (%)**	66.2	66.4	63.3	**0.7**
**Parental history of CVD (%)**	51.9	51.4	58.3	**0.4**
**Negative Moods**				
**Anger in (median [IQR])**	5 [3–7]	5 [3–7]	5 [3–7]	**0.7**
Mean (SD)	5.2 (3.4)	5.2 (3.5)	5.1 (2.9)	
**Anger out (median [IQR])**	4 [2–6]	4 [2–6]	5 [3–7]	**0.1**
Mean (SD)	4.3 (3.0)	4.3 (3.0)	4.9 (2.9)	
**Hostility (median [IQR])**	11 [8–15]	11 [8–15]	12 [8–15]	**0.4**
Mean (SD)	11.5 (4.8)	11.5 (4.7)	12.0 (5.0)	
**Religiosity**				
**Spirituality (median [IQR])**	24 [21–27]	24 [21–27]	24 [23–26]	**0.02**
Mean (SD)	23.3 (4.8)	23.2 (4.9)	24.3 (3.4)	
**Organized religion (median [IQR])**	4 [4–5]	4 [4–5]	4 [4–5]	**0.01**
Mean (SD)	4.03 (0.9)	4.01 (0.9)	4.3 (0.7)	
**Non-organized religion (median [IQR])**	7 [6–7]	7 [6–7]	7 [6–7]	**0.07**
Mean (SD)	6.3 (1.2)	6.3 (1.2)	6.5 (1.0)	
**Religious coping (median [IQR])**	3 [2–3]	3 [2–3]	3 [2–3]	**0.7**
Mean (SD)	2.6 (0.7)	2.6 (0.7)	2.6 (0.8)	
**Discrimination**				
**Daily discrimination (median [IQR])**	7 [3–14]	7 [3–14]	8 [3–14]	**0.4**
Mean (SD)	9.1 (8.5)	9.1 (8.4)	10.2 (9.7)	
**Lifetime discrimination (median [IQR])**	3 [2–4]	3 [2–5]	3 [1–4]	**0.1**
Mean (SD)	3.2 (2.1)	3.2 (2.1)	2.8 (1.9)	
**Burden of lifetime discrimination (median [IQR])**	3.5 [2–5.5]	3.5 [2–5.5]	3.5 [1.9–5.1]	**0.6**
Mean (SD)	3.8 (2.4)	3.8 (2.4)	3.7 (2.3)	
**Stress (median [IQR])**	4 [2–8]	4 [2–8]	4 [1.8–7.3]	**0.5**
Mean (SD)	5.0 (4.3)	5.1 (4.3)	4.7 (4.0)	
**Negative Outlook**				
**Pessimism (median [IQR])**	2 [1–5]	2 [1–5]	3 [1–7]	**0.02**
Mean (SD)	3.3 (3.0)	3.2 (3.0)	4.2 (3.0)	
**John Henryism (median [IQR])**	30 [27–33]	30 [27–33]	31 [27–33]	**0.7**
Mean (SD)	29.7 (4.3)	29.7 (4.3)	30.0 (4.6)	
**Negative coping resources**				
**Social support (median [IQR])**	6 [4–8]	6 [4–8]	6 [3–8]	**0.7**
Mean (SD)	6.0 (2.6)	6.0 (2.6)	5.9 (2.9)	
**Social status (median [IQR])**	3 [2–5]	3 [2–5]	3 [2–5]	**0.4**
Mean (SD)	3.4 (2.0)	3.4 (2.0)	3.6 (2.0)	

*SD* standard deviation

*IQR* interquartile range

*CVD* cardiovascular disease

### Principal component analysis

**Fig. 2:**
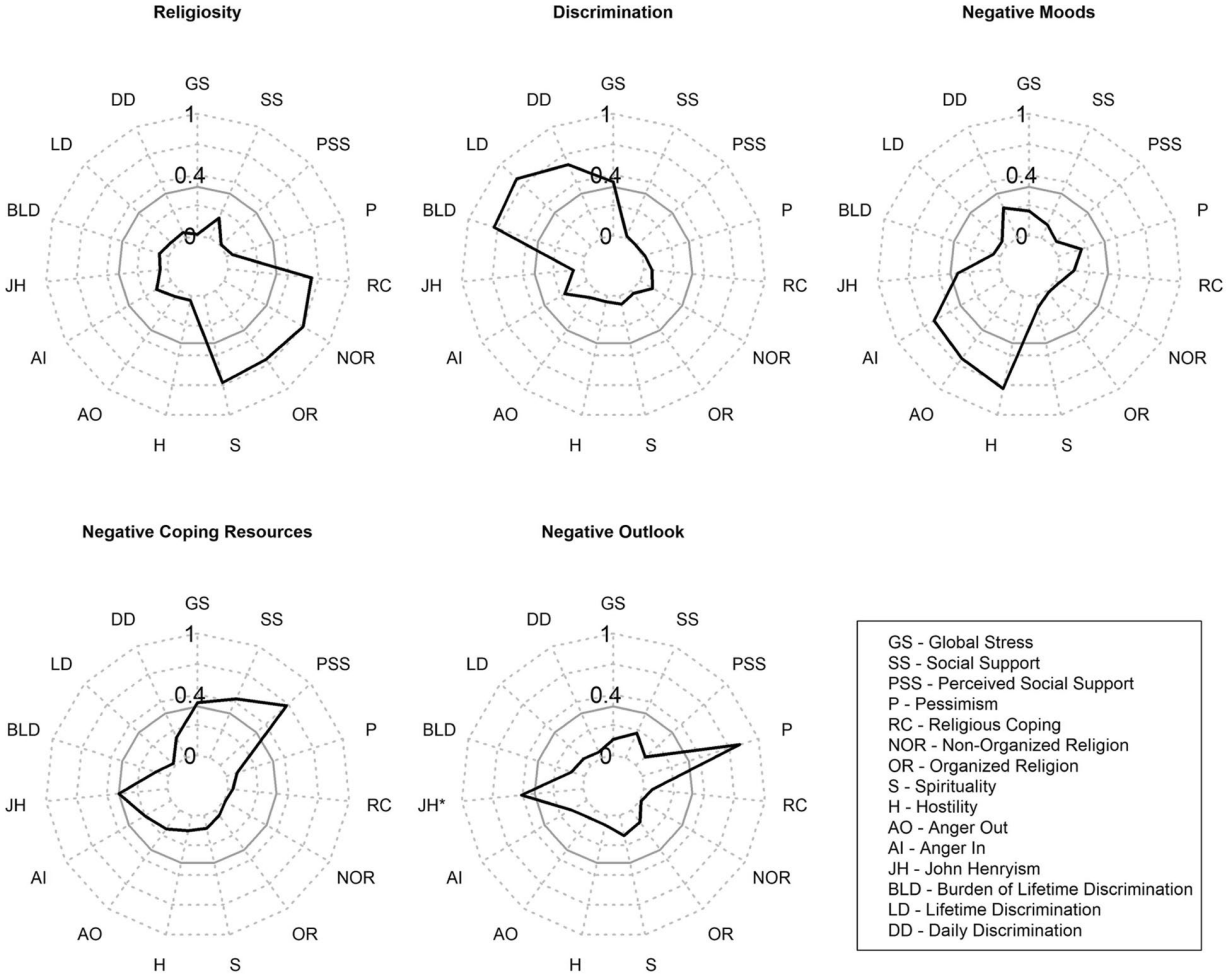
Radar plots showing variables that load on each component

PCA identified five rotated components representing constructs related to psychosocial health (Fig. 2). We labeled these components “negative moods,” “religiosity,” “discrimination,” “negative outlook,” and “negative coping resources.” Negative moods comprised anger in, anger out, and hostility. Religiosity was composed of constructs representing spirituality, organized religion, non-organized religion, and religious coping. Discrimination comprised perceived discrimination and stress. Negative outlooks consisted of John Henryism and pessimism. Negative coping resources comprised perceived social status and social support. For most components, higher scores represented higher psychosocial levels. For negative coping resources, perceived social status and social support were reverse scored so that higher scores indicate lower social standing and less social support.

### Association of psychosocial components with CVD events

During a median follow-up of 8 years, 6% (*n* = 60) of participants had an incident CVD event. After adjustment, each standard deviation increase in negative moods was associated with a non-statistically significantly greater hazard for CVD events (HR = 1.26, 95% CI: 0.97–1.65) (Table 2). The hazard ratio for CVD was greater among participants with negative mood scores above the median (HR = 1.62; 95% CI: 0.92, 2.85) and in the highest tertile (HR = 1.90; 95% CI: 0.93, 3.87) as compared to scores below the median and in the lowest tertile, respectively, however these associations were not statistically significant. Each standard deviation increase in the religiosity component—representing greater levels of religiosity—was associated with a statistically significantly greater hazard for CVD events (HR = 1.52, 95% CI: 1.09–2.13). This association persisted when evaluating dichotomized cohort medians (HR = 1.94, 95% CI: 1.10–3.39). Discrimination, negative coping resources, and negative outlook were not significantly associated with CVD events in this cohort after adjusting for other covariates. There was no statistically significant interaction between these psychosocial components and CVD (Table 3).

**Table 2:** Hazard ratio for the association between continuous and categorical components and risk of incident CVD

No. of events/N: Imputed: 60/929	Per SD	Above/Below Median	Tertile of Component Score		
Component Name			T1	T2	T3
**Negative Moods**	1.26 (0.97, 1.65)	1.62 (0.92, 2.85)	1 (REF)	1.64 (0.81, 3.32)	1.9 (0.93, 3.87)
**Religiosity**	1.52 (1.09, 2.13)	1.94 (1.1, 3.39)	1 (REF)	2.01 (0.99, 4.07)	1.9 (0.92, 3.9)
**Discrimination**	0.86 (0.65, 1.14)	0.95 (0.56, 1.61)	1 (REF)	0.93 (0.49, 1.75)	0.74 (0.38, 1.43)
**Negative Outlook**	1.14 (0.88, 1.48)	1.27 (0.73, 2.19)	1 (REF)	1.03 (0.5, 2.09)	1.31 (0.68, 2.54)
**Negative coping resources**	1.24 (0.93, 1.64)	1.47 (0.85, 2.55)	1 (REF)	1.52 (0.79, 2.94)	1.62 (0.81, 3.25)

Models were adjusted for age, sex, diabetes status, dyslipidemia, hypertension, antihypertensive medication, chronic kidney disease, smoking status, body mass index, family history of CVD, education, income, and employment

**Table 3:** Age and sex stratified hazard ratio for association between SD increase in component and risk of CVD

	Age		Sex	
No. of events/N Imputed: 60/929	< 57 years 18	≥ 57 years 42	Male 26	Female 34
**Negative Moods**	1.43 (0.88, 2.33)	1.25 (0.90, 1.73)	1.39 (0.85, 2.27)	1.26 (0.89, 1.79)
**Religiosity**	1.60 (0.90, 2.83)	1.49 (0.96, 2.31)	1.76 (1.07, 2.90)	1.35 (0.79, 2.31)
**Discrimination**	1.25 (0.74, 2.12)	0.85 (0.60, 1.21)	0.60 (0.38, 0.96)	0.97 (0.66, 1.41)
**Negative Outlook**	0.92 (0.47, 1.80)	1.25 (0.92, 1.71)	1.48 (0.93, 2.34)	1.07 (0.74, 1.55)
**Negative coping resources**	1.21 (0.7, 2.08)	1.16 (0.79, 1.70)	1.18 (0.74, 1.89)	1.24 (0.86, 1.81)

All models were adjusted for age, sex, diabetes status, dyslipidemia, hypertension, antihypertensive medication, chronic kidney disease, smoking status, body mass index, family history of CVD, education, income, and employment

### Sensitivity analyses

#### Rotated principle component analysis

In sensitivity analyses, stress loaded on the negative coping resources component. The discrimination component, therefore, included only the perceived discrimination variables while the negative coping resources components included perceived social status, social support, and stress (Additional file 1: Appendix 3).

#### Missing CKD status

To assess the impact of missing CKD status on results, models were re-fit to include 390 participants with missing CKD status. Excluded individuals tended to be older, less educated, lower income, less likely to be working for wages, either current or former smokers, and engaged in lower levels of physical activity. We did not see significant differences in clinical characteristics such as antihypertensive medication use, dyslipidemia, or parental history of CVD (Additional file 1: Appendix 4). Results were attenuated and, for most associations, had similar direction of effect as in main analyses. Of note, participants in the highest tertile of discrimination had a lower hazard of CVD compared to participants in the lowest tertile of discrimination (HR = 0.56; 95% CI: 0.32, 0.95) (Table 4). Among older participants (≥ 58 years), each SD increase in discrimination score was associated with a lower hazard for CVD (HR = 0.70; 95% CI: 0.53, 0.94). For each increase in standard deviation of religiosity, male participants had marginally higher hazard of CVD (HR: 1.40; 95% CI: 0.96, 2.06) (p-interaction = 0.06) (Table 5).

**Table 4:** Hazard ratio for association between continuous and categorical component and risk of incident CVD when including participants with missing CKD status

No. of events/N: Imputed: 97/1319	Per SD	Above/Below Median	Tertile of Component Score		
Component Name			T1	T2	T3
**Negative Moods**	1.10 (0.88, 1.37)	1.02 (0.67, 1.57)	1 (REF)	1.17 (0.69, 1.97)	1.28 (0.75, 2.19)
**Religiosity**	1.13 (0.89, 1.44)	1.27 (0.83, 1.95)	1 (REF)	1.10 (0.65, 1.86)	1.19 (0.69, 2.05)
**Discrimination**	0.80 (0.64, 1.01)	0.75 (0.49, 1.15)	1 (REF)	0.75 (0.47, 1.2)	0.56 (0.32, 0.95)
Negative Outlook*	1.11 (0.91, 1.37)	0.99 (0.65, 1.51)	1 (REF)	1.1 (0.65, 1.88)	1.2 (0.71, 2.01)
Negative coping resources*	1.11 (0.91, 1.37)	1.26 (0.83, 1.91)	1 (REF)	1.26 (0.76, 2.08)	1.39 (0.83, 2.32)

All models were adjusted for age, sex, diabetes status, dyslipidemia, hypertension, antihypertensive medication, chronic kidney disease, smoking status, body mass index, family history of CVD, education, income, and employment

* In the main analysis, John Henryism loaded on Negative Outlook. In analysis that includes participants with missing CKD status, John Henryism loads on Negative Coping Resources

**Table 5:** Age and sex stratified hazard ratio for association between SD increase in component and risk of incident CVD when including participants with missing CKD status

	Age		Sex	
No. of Events	< 58 years 29	≥ 58 years 68	Male 36	Female 61
**Negative Moods**	1.31 (0.9, 1.89)	1.03 (0.79, 1.35)	1.29 (0.88, 1.9)	1.01 (0.77, 1.33)
**Religiosity**	1.18 (0.78, 1.79)	1.05 (0.78, 1.4)	1.4 (0.96, 2.06)	0.9 (0.64, 1.25)
**Discrimination**	1.01 (0.69, 1.48)	0.7 (0.53, 0.94)	0.75 (0.51, 1.11)	0.8 (0.59, 1.07)
Negative Outlook*	0.92 (0.61, 1.38)	1.22 (0.95, 1.56)	1.1 (0.76, 1.6)	1.09 (0.84, 1.41)
Negative coping resources*	1.36 (0.95, 1.95)	0.97 (0.74, 1.27)	1.06 (0.74, 1.54)	1.13 (0.87, 1.48)

All models were adjusted for age, sex, diabetes status, dyslipidemia, hypertension, antihypertensive medication, chronic kidney disease, smoking status, body mass index, family history of CVD, education, income, and employment

* In the main analysis, John Henryism loaded on Negative Outlook. In analysis that includes participants with missing CKD status, John Henryism loads on Negative Coping Resources

#### Complete case analysis

Sensitivity analyses were conducted using individuals with complete covariate data. These individuals were more likely to be female (Additional file 1: Appendix 5: 70%) as compared to individuals with some missing covariate data (Table 1: 67%). Other baseline characteristics were largely similar between groups (Additional file 1: Appendix 5/Table 1). The association between psychosocial components and incident CVD was largely similar in analyses where data were imputed as compared to complete case analyses (Additional file 1: Appendix 6–8). Associations calculated in complete case analysis tended to be further away from the null value of 0 as compared to associations calculated using imputed data.

#### HDL as a covariate

Inclusion of HDL as a covariate did not appreciably change the results (data not shown).

## Discussion

In this community-based study of Black adults at risk for CKD and CKD risk factors, PCA – a form of unsupervised machine learning – identified three components (religiosity, discrimination, and negative moods) that a person would be likely to group together and two other components (negative coping resources and pessimism) that may not be intuitively combined by a person. Higher levels of religiosity were significantly associated with greater risk for CVD events. Taken together, these results suggest that the impact of psychosocial components on CVD is complex and nuanced.

Our findings add to growing evidence of the association of religiosity with CVD and align with several prior studies. In a study of 92,395 women in the Women’s Health Initiative Observational Study, religious affiliation was associated with increased risk for coronary heart disease morbidity, and mortality in models adjusted for demographic, socioeconomic, clinical, psychosocial, and health behavior factors [10]. In this same study, women who stated that religion provides “a little” or “a great deal” of strength and comfort as compared to women who stated that religion provides no strength or comfort were at increased risk for CVD morbidity and mortality. In a separate study of 5474 White, Black, Hispanic, and Chinese participants from the Multi-ethnic Study of Atherosclerosis (MESA), individuals who participated in religious practices weekly or daily were at increased risk for sub-clinical CVD as measured by common carotid intima media thickness [11]. This study did not find a significant positive association between religious practice and other sub-clinical measures of CVD. Our results support these latter studies in that we found a positive association between religiosity and CVD. There is considerably less research on the mechanisms that promote an association between greater levels of religiosity and increased risk for adverse health outcomes. Black adults are more likely to use religion and spirituality as coping mechanisms when confronted with health issues compared to other groups [32, 33]. Thus, our observation about religiosity and CVD may underscore broader relationships between psychosocial stress, coping, and CVD. In our study, religiosity was high in the at-risk population, which may impact the direction of association as the association we saw may represent a ceiling effect on the outcome. That is, the variance in religiosity in this population may be relatively small because religiosity is high compared to the general population. Individuals participating in religious activities could also have differential behaviors that impact CVD risk, such as diet or patterns of physical activity. Additionally, religiosity may be a form of coping with risk of CVD. Recent studies have suggested that variations in coping strategies influence health risk [34].

Our results on religiosity also conflict with many prior studies. For instance, multiple studies have reported that religiosity and spiritualty are protective factors for various physical and mental health outcomes, including heart disease, hypertension, stroke, end stage kidney disease, cancer, gastrointestinal disease, depression, and anxiety [35, 36]. The mechanisms that drive these salutary associations are not completely clear, but it has been proposed that they include greater social support, healthier lifestyles, and improved psychological and biochemical markers [35, 37–42]. Religious institutions themselves often serve as central meeting places for people to engage in supportive and community-building activities [43]. These social interactions may help individuals’ abilities to cope with stressful circumstances [44]. They may also improve life satisfaction, optimism, and self-esteem, which, in turn, may improve a person’s ability to respond to adversity (i.e., increase resilience) [45, 46]. Religious institutions could also promote healthy behaviors as many religions discourage tobacco and alcohol consumption, drug use, and risky sexual behavior [47–49]. These positive health behaviors can result in lower blood pressure, improved cardiac ability, and less bodily inflammation, resulting in lower risk for precursors of CVD events. A smaller number of studies have reported that religiosity and spirituality are not protective for health outcomes [10, 11] or that there is a salutary association of religiosity and health outcomes [50, 51]. The baseline risk profile of our population describes a relatively high-risk group which may have impact the higher CVD risk seen among individuals with greater levels of religiosity. Additional research on the association between religiosity and health outcomes may consider including more granular details about how other behaviors central to specific religions (e.g., abstaining from alcohol, vegetarian diets) may impact risk for health outcomes.

We also found a positive (albeit non-significant) trend in the association between negative moods (i.e., anger and hostility) and increased risk of CVD events. This aligns with prior research among the general population where anger and hostility have been shown to be independent risk factors for CVD [52, 53]. Higher levels of discrimination – which included stress – were associated with marginally decreased risk of CVD in our study. This unexpected result is supported by prior research in the JHS [30]. Sims et al. found that men participating in the JHS with higher levels of lifetime and everyday discrimination had lower high-sensitivity C-reactive protein levels. As noted in their study, this finding may be the result of the relatively small male population in the study and the fact that the JHS male cohort – that is, older, lower SES, and Black – are underrepresented in epidemiologic research [54, 55]. Other studies of stress and CVD have focused on employment-related stressors and noted that high work-related effort with low job control/rewards may negatively impact cardiovascular health [56]. Less has been published on the association between composite measures of stress and CVD. The Multiple Risk Factor Intervention Trial study did report an increased risk for CVD mortality related to work and marital stressors [57]. There are potentially multiple explanations for these findings. Studies of the association between anger/hostility and stress/social support and CVD have mainly reported findings on white men. Our study included Black men and women, in whom the association between anger/hostility and stressors and CVD may differ from that of white men and women. Also, we used a composite measure of discrimination summarized through PCA, including measures for stress, employment, relationships, neighborhood, caring for others, legal problems, medical problems, racism and discrimination, and meeting basic needs. This composite measure captures the synergistic effect of these moods/stressors on cardiovascular health. Perhaps this composite represents a more real-world cumulative depiction of how moods/stress impact CVD among Black adults.

Our study did have limitations. The JHS is restricted to a Black population in a distinct geographic region around Jackson, Mississippi, limiting the generalizability of our findings in Black adults outside of Jackson MS. Also, psychosocial factors were only assessed at baseline within our study, and they may not reflect changes that occur over time. Future work may want to explore the impact of longitudinal (or time-varying) measures of psychosocial factors on risk of CVD. The rotated PCA we conducted has inherent limitations. We applied heuristics to the derived components; heuristics are subjective and different heuristics can be applied to these components by different people. In sensitivity analysis that included participants with missing CKD information or covariate information, associations were attenuated, albeit in a similar direction to the main associations, suggesting that those with missing CKD or covariate information may be at lower risk for CVD. The relatively small number of outcomes [58, 59] limited our ability to conduct subgroup analyses but were in line with prior studies that use JHS data and other cohorts. In two studies using participants from the Atherosclerosis Risk in Communities (ARIC) study, CVD developed in 19–26% of the Black participants over a median of 8.5–11 years. Of note, CVD was defined here as probable or definite stroke, coronary heart disease (CHD), defined as myocardial infarction, revascularization procedure or congestive heart failure (CHF). CHF was not included as an outcome in our study. The ARIC study enrolled participants prior to statins becoming widely available and there have been numerous advances in CVD treatment. A more comparable population may be in the Reasons for Geographic and Racial Differences in Stroke (REGARDS) study; baseline information was collected from 2003 to 2007 [59]. Participants included 10,348 black individuals with a mean age of 64 years. Over a 4-year follow-up period, 2.8% of the black participants developed CHD. In a study that looked at the risk for CVD among JHS participants with prediabetes and/or hypertension over a mean of 11 years, 3–15% of participants developed CVD, defined as probably or definite CHD, stroke, or CHF [58]. If CVD was defined only by CHD and/or stroke (as we did in our study), the proportion developing CVD ranged from 2 to 11%. Notwithstanding these limitations, the JHS is the largest study of CVD within African Americans and is unique in its robust, validated measures of psychosocial factors. Additionally, this study has a long follow-up period with adjudicated outcomes.

## Conclusions

In conclusion, we observed that increased religiosity was associated with greater risk of CVD events among Black adults with CKD or CKD risk factors. Future studies are needed to investigate potential social and biological mechanisms for these associations and to develop and evaluate potential targeted interventions.

## Supplementary Information

**Additional file 1: Appendix 1**: Number of unique participants with 0, 1, 2, 3 missing variables. **Appendix 2**: Summary of variables missing. **Appendix 3**. Radar plots showing variables that load on each component for sensitivity analysis. **Appendix 4**: Characteristics of participants included and excluded in the analysis at baseline and follow-up. **Appendix 5**: Baseline characteristics of the study population used in complete case analysis. **Appendix 6**. Complete case analysis of the association between continuous and categorical component scores and risk of incident CVD. **Appendix 7**. Complete case analysis of age and sex stratified association between SD increase in component score and risk of CVD. **Appendix 8a**. Complete case analysis for association between continuous and categorical component and risk of incident CVD when including participants with missing CKD status. **Appendix 8b**. Complete case analysis for age and sex stratified association between SD increase in component and risk of incident CVD when including participants with missing CKD status.

## Data Availability

The data that support the findings of this study are available from The Jackson Heart Study but restrictions apply to the availability of these data, which were used under license for the current study, and so are not publicly available. The JHS data are available to researchers with approved manuscript proposals. The JHS data and materials can be requested from the JHS Committee at https://www.jacksonheartstudy.org/Research/Study-Data/Data-Access.

## References

[1] Saran R, Robinson B, Abbott KC, et al. US Renal Data System Annual Data Report: Epidemiology of Kidney Disease in the United States. AJKD, 2019;69(3 Suppl 1):A7–A8. 10.1053/j.ajkd.2016.12.004 PMC6605045 28236831

[2] Sarnak MJ, Levey AS, Schoolwerth AC (2003). Kidney disease as a risk factor for development of cardiovascular disease: a statement from the American Heart Association councils on kidney in cardiovascular disease, high blood pressure research, clinical cardiology, and epidemiology and prevention. Circulation.

[3] Mehrotra R, Kermah D, Fried L, Adler S, Norris K (2008). Racial differences in mortality among those with CKD. J Am Soc Nephrol.

[4] Everson-Rose SA, Lewis TT (2005). Psychosocial factors and cardiovascular diseases. Annu Rev Public Health.

[5] Sims M, Diez-Roux AV, Dudley A (2012). Perceived discrimination and hypertension among African Americans in the Jackson heart study. Am J Public Health.

[6] Williams DR, Neighbors H (2001). Racism, discrimination and hypertension: evidence and needed research. Ethn Dis.

[7] Kendzor DE, Businelle MS, Mazas CA (2009). Pathways between socioeconomic status and modifiable risk factors among African American smokers. J Behav Med.

[8] Lee C, Ayers SL, Kronenfeld JJ (2009). The association between perceived provider discrimination, healthcare utilization and health status in racial and ethnic minorities. Ethn Dis.

[9] Chida Y, Steptoe A (2009). The association of anger and hostility with future coronary heart disease: a meta-analytic review of prospective evidence. J Am Coll Cardiol.

[10] Schnall E, Wassertheil-Smoller S, Swencionis C (2010). The relationship between religion and cardiovascular outcomes and all-cause mortality in the Women's Health Initiative observational study. Psychol Health.

[11] Feinstein M, Liu K, Ning H, Fitchett G, Lloyd-Jones DM (2010). Burden of cardiovascular risk factors, subclinical atherosclerosis, and incident cardiovascular events across dimensions of religiosity: the multi-ethnic study of atherosclerosis. Circulation.

[12] Pascoe EA, Smart RL (2009). Perceived discrimination and health: a meta-analytic review. Psychol Bull.

[13] Clark R, Anderson NB, Clark VR, Williams DR (1999). Racism as a stressor for African Americans. A biopsychosocial model. Am Psychol.

[14] Borrell LN, Kiefe CI, Diez-Roux AV, Williams DR, Gordon-Larsen P (2013). Racial discrimination, racial/ethnic segregation, and health behaviors in the CARDIA study. Ethn Health.

[15] Sims M, Glover LSM, Gebreab SY, Spruill TM (2020). Cumulative psychosocial factors are associated with cardiovascular disease risk factors and management among African Americans in the Jackson heart study. BMC Public Health.

[16] Fuqua SR, Wyatt SB, Andrew ME (2005). Recruiting African-American research participation in the Jackson Heart Study: methods, response rates, and sample description. Ethn Dis.

[17] Levey AS, Stevens LA, Schmid CH (2009). A new equation to estimate glomerular filtration rate. Ann Intern Med.

[18] Williams DR, Yan Y, Jackson JS, Anderson NB (1997). Racial differences in physical and mental health: socio-economic status, stress and discrimination. J Health Psychol.

[19] Payne TJ, Wyatt SB, Mosley TH (2005). Sociocultural methods in the Jackson Heart Study: conceptual and descriptive overview. Ethn Dis.

[20] James SA, Jones RL (1996). The John Henryism scale for active coping. Handbook of tests and measurements for black populations.

[21] Underwood LG, Teresi JA (2002). The daily spiritual experience scale: development, theoretical description, reliability, exploratory factor analysis, and preliminary construct validity using health-related data. Ann Behav Med.

[22] Barefoot JC, Dodge KA, Peterson BL, Dahlstrom WG, Williams RB (1989). The cook-medley hostility scale: item content and ability to predict survival. Psychosom Med.

[23] Spielberger CD, Gorsuch RL, Lushene R, Vagg PR, Jacobs GA (1983). Manual for the state-trait anxiety inventory.

[24] Carver CS, Scheier MF, Segerstrom SC (2010). Optimism. Clin Psychol Rev.

[25] Lubben J (1988). Assessing social networks among elderly populations. Fam Commun Health.

[26] Revelle W (2021). Psych: procedures for psychological, psychometric, and personality research. Northwestern University.

[27] Richman M (1986). Rotation of principal components. Int J Climatol.

[28] van Buuren S, Groothuis-Oudshoorn CGM. mice: Multivariate Imputation by Chained Equations in R. J Stat Softw. 2011;45(3). http://www.jstatsoft.org/v45/i03.

[29] Sims M, Lipford KJ, Patel N, Ford CD, Min YI, Wyatt SB (2017). Psychosocial factors and behaviors in African Americans: the Jackson heart study. Am J Prev Med.

[30] Dunlay SM, Lippmann SJ, Greiner MA (2017). Perceived discrimination and cardiovascular outcomes in older African Americans: insights from the Jackson heart study. Mayo Clin Proc.

[31] Fine JP, Gray RJ (1999). A proportional hazards model for the subdistribution of a competing risk. J Am Stat Assoc.

[32] Koenig HG, George LK, Cohen HJ, Hays JC, Larson DB, Blazer DG (1998). The relationship between religious activities and cigarette smoking in older adults. J Gerontol A Biol Sci Med Sci.

[33] Mansfield CJ, Mitchell J, King DE (2002). The doctor as God's mechanic? Beliefs in the southeastern United States. Soc Sci Med.

[34] Brenner AB, Diez-Roux AV, Gebreab SY, Schulz AJ, Sims M (2018). The epidemiology of coping in African American adults in the Jackson heart study (JHS). J Racial Ethn Health Disparities.

[35] Koenig HG, Hays JC, Larson DB (1999). Does religious attendance prolong survival? A six-year follow-up study of 3,968 older adults. J Gerontol A Biol Sci Med Sci.

[36] Nair D, Cavanaugh KL, Wallston KA (2020). Religion, spirituality, and risk of end-stage kidney disease among adults of low socioeconomic status in the southeastern United States. J Health Care Poor Underserved.

[37] Ellison CG, Levin JS (1998). The religion-health connection: evidence, theory, and future directions. Health Educ Behav.

[38] Koenig HG (2001). Religion and medicine III: developing a theoretical model. Int J Psychiatry Med.

[39] Larson DB, Sherrill KA, Lyons JS (1992). Associations between dimensions of religious commitment and mental health reported in the American journal of psychiatry and archives of general psychiatry: 1978-1989. Am J Psychiatry.

[40] Roff LLKD, Parker M, Koenig HG, Sawyer-Baker P, Allman RM (2005). Religiosity, smoking, exercise, and obesity among southern, community dwelling older adults. J Appl Gerontol.

[41] Strawbridge WJ, Cohen RD, Shema SJ, Kaplan GA (1997). Frequent attendance at religious services and mortality over 28 years. Am J Public Health.

[42] Koenig HG (1999). The healing power of faith.

[43] Wilson J, Musick M (1997). Who Cares? Toward an Integrated Theory of Volunteer Work. Am Sociol Rev..

[44] Krause N (2011). Religion and health: making sense of a disheveled literature. J Relig Health.

[45] Krause N, Ellison CG, Shaw BA, Marcum JP, Boardman JD (2001). Church based social support and religious coping. J Sci Study Relig..

[46] Pargament KI, Koenig HG, Perez LM (2000). The many methods of religious coping: development and initial validation of the RCOPE. J Clin Psychol.

[47] Powell LH, Shahabi L, Thoresen CE (2003). Religion and spirituality. Linkages to physical health. Am Psychol.

[48] Strawbridge WJ, Shema SJ, Cohen RD, Kaplan GA (2001). Religious attendance increases survival by improving and maintaining good health behaviors, mental health, and social relationships. Ann Behav Med.

[49] Thege BKPJ, Szekely A, Kopp MS (2003). Relationship between religiosity and health: evidence from a post-communist country. Int J Behav Med..

[50] Levin JS, Schiller PL (1987). Is there a religious factor in health?. J Relig Health.

[51] Jarvis GK, Northcott HC (1987). Religion and differences in morbidity and mortality. Soc Sci Med.

[52] Matthews KA, Gump BB, Harris KF, Haney TL, Barefoot JC (2004). Hostile behaviors predict cardiovascular mortality among men enrolled in the multiple risk factor intervention trial. Circulation.

[53] Williams JE, Nieto FJ, Sanford CP, Couper DJ, Tyroler HA (2002). The association between trait anger and incident stroke risk: the atherosclerosis risk in communities (ARIC) study. Stroke.

[54] Sims KD, Sims M, Glover LM, Smit E, Odden MC (2020). Perceived discrimination and trajectories of C-reactive protein: the Jackson heart study. Am J Prev Med.

[55] Webb FJ, Khubchandani J, Striley CW, Cottler LB (2019). Black-white differences in willingness to participate and perceptions about Health Research: results from the population-based HealthStreet study. J Immigr Minor Health.

[56] Siegrist J, Starke D, Chandola T (2004). The measurement of effort-reward imbalance at work: European comparisons. Soc Sci Med.

[57] Matthews KA, Gump BB (2002). Chronic work stress and marital dissolution increase risk of posttrial mortality in men from the multiple risk factor intervention trial. Arch Intern Med.

[58] Hubbard D, Colantonio LD, Tanner RM (2019). Prediabetes and risk for cardiovascular disease by hypertension status in black adults: the Jackson heart study. Diabetes Care.

[59] Safford MM, Brown TM, Muntner PM (2012). Association of race and sex with risk of incident acute coronary heart disease events. JAMA.

