# Supplementary material for: Psychosocial determinants of cardiovascular events among black Americans with chronic kidney disease or associated risk factors in the Jackson heart study
Source: BMC Nephrol. 2021 Nov 11;22:375. doi: 10.1186/s12882-021-02594-6 (PMC8582093; doi:10.1186/s12882-021-02594-6)
Supplement: Supplementary file 1 — Additional file 1: Appendix 1: Number of unique participants with 0, 1, 2, 3 missing variables. Appendix 2: Summary of variables missing. Appendix 3. Radar plots showing variables that load on each component for sensitivity analysis. Appendix 4: Characteristics of participants included and excluded in the analysis at baseline and follow-up. Appendix 5: Baseline characteristics of the study population used in complete case analysis. Appendix 6. Complete case analysis of the association between continuous and categorical component scores and risk of incident CVD. Appendix 7. Complete case analysis of age and sex stratified association between SD increase in component score and risk of CVD. Appendix 8a. Complete case analysis for association between continuous and categorical component and risk of incident CVD when including participants with missing CKD status. Appendix 8b. Complete case analysis for age and sex stratified association between SD increase in component and risk of incident CVD when including participants with missing CKD status. [file 12882_2021_2594_MOESM1_ESM.docx]

**Appendix 1: Number of unique participants with 0, 1, 2, 3 missing variables**

| **# of Missing Variables** | **0** | **1** | **2** | **3** | **Total** |
| --- | --- | --- | --- | --- | --- |
| **# of Participants** | **609** | **248** | **65** | **7** | **929** |

**Appendix 2: Summary of variables missing (N=929)**

| **Variable** | **N Missing** | **Percent Missing** |
| --- | --- | --- |
| Income | 148 | 15.9 |
| Dyslipidemia | 88 | 9.5 |
| Nutrition | 72 | 7.8 |
| Antihypertensive medication use | 60 | 6.5 |
| Depression | 21 | 2.3 |
| Employment status | 3 | 0.3 |
| Alcohol use | 2 | 0.2 |
| Diabetes | 1 | 0.1 |
| Systolic Blood Pressure | 1 | 0.1 |
| Diastolic Blood Pressure | 1 | 0.1 |
| BMI | 1 | 0.1 |
| Parental History of CVD | 1 | 0.1 |

**Appendix 3. Radar plots showing variables that load on each component for sensitivity analysis**

**
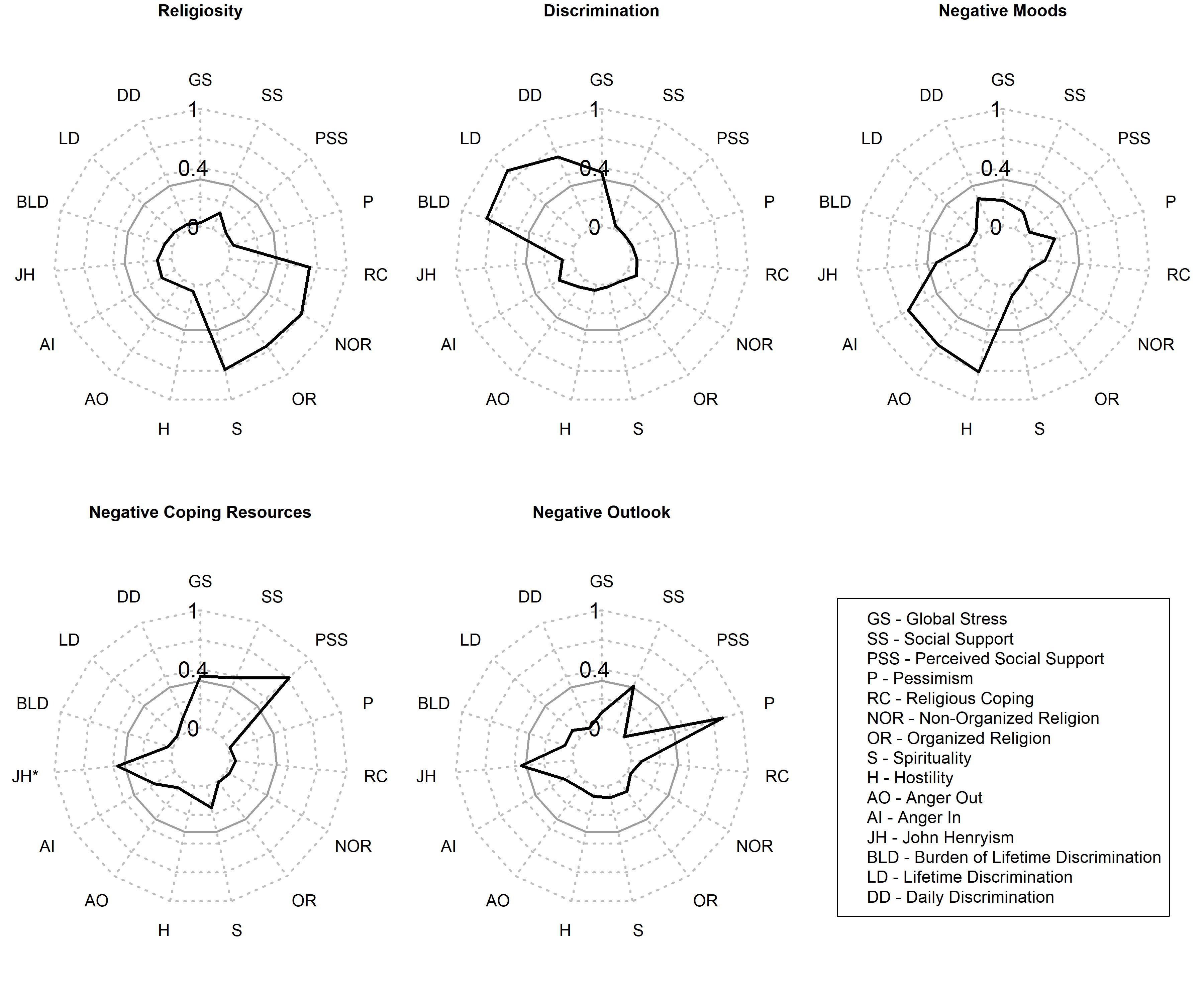
**

Variables used to define components are on the perimeter of the radar plots (abbreviations defined in figure legend).

Concentric circles (dashed gray) represent absolute value of factor loadings; solid gray circle represent the threshold (0.4) to signify a variable that loads on a component.

Of note, perceived social status and social support are reverse coded.

**Appendix 4: Characteristics of participants included and excluded in the analysis at baseline and follow-up.**

|  | **Main analysis (before imputation)** | | **Excluded due to missing psychosocial data** | **Participants with missing baseline CKD status** |
| --- | --- | --- | --- | --- |
| **n** | 929 | | 990 | 390 |
| **Age, years (mean, SD)** | 56.4 (11.4) | | 58.6 (12.7) | 58.6 (9.5) |
| **Female (%)** | 66.6 | | 63.0 | 72.1 |
| **Education (%)** |  | |  |  |
| **<High school** | 30.8 | | 46.2 | 38.2 |
| **≥High school** | 69.2 | | 53.3 | 61.5 |
| **Missing** | 0 | | 0.51 | 0.26 |
| **Income (%)** |  | |  |  |
| **≤1.5x poverty level** | 27.1 | | 33.5 | 34.6 |
| **>1.5x poverty level** | 56.9 | | 47.3 | 55.4 |
| **Missing** | 15.9 | | 19.2 | 10 |
| **Employment (%)** |  | |  |  |
| **Not working for wages** | 39.1 | | 43.0 | 39.7 |
| **Working for wages** | 60.9 | | 56.5 | 59.2 |
| **Missing** | 0 | | 0.51 | 1.03 |
| **Body mass index** | 32.8 (7.2) | | 32.3 (7.3) | 32.6 (6.6) |
| **Missing** | 0 | | 0.2 | 0.2 |
| **Smoking status (%)** |  | |  |  |
| **Former/current** | 27.8 | | 31.4 | 36.4 |
| **Missing** | 0 | | 0.2 | 0.26 |
| **Antihypertensive medication use (%)** | 75.6 | | 74.7 | 77.9 |
| **Missing** | 6.5 | | 9.9 | 7.4 |
| **Dyslipidemia (%)** | 58.8 | | 55.3 | 57.9 |
| **Missing** | 9.5 | | 15.3 | 9.7 |
| **Parental history of CVD (%)** | 51.9 | | 52 | 50.5 |
| **Missing** | 0.1 | | 0.1 | 0 |
| **Alcohol use (%)** | 41.6 | | 37.6 | 44.4 |
| **Missing** | 0.2 | | 0.6 | 0.3 |
| **Nutrition (%)** |  | |  |  |
| **Poor** | 48.1 | | 46 | 47.4 |
| **Intermediate** | 42.9 | | 44 | 38.7 |
| **Ideal** | 1.2 | | 1.1 | 1 |
| **Missing** | 7.8 | | 8.9 | 12.8 |
| **Physical Activity (%)** |  | |  |  |
| **Poor** | 46.8 | | 53.5 | 54.6 |
| **Intermediate** | 33.2 | | 30.5 | 27.4 |
| **Ideal** | 20 | | 15.9 | 17.9 |
| **Missing** | 0 | | 0.1 | 0 |
| **Depression (%)** | 45.5 | | 18.2 | 47.2 |
| **Missing** | | 2.3 | 59.9 | 1.5 |

CVD = cardiovascular disease

**Appendix 5: Baseline characteristics of the study population used in complete case analysis**

|  | **Overall** | **No CVD** | **Incident CVD** | **p-value** |
| --- | --- | --- | --- | --- |
| **n** | 609 | 563 | 46 |  |
| **Age, years (mean ± SD)** | 56.8 (11.2) | 56.5 (11.1) | 61.5 (11.6) | p=0.01 |
| **Female (%)** | 70.4 | 71.2 | 60.9 | 0.2 |
| **Education (%)** |  |  |  | 0.3 |
| <High school | 28.6 | 27.9 | 37 |  |
| ≥High school | 71.4 | 72.1 | 63 |  |
| **Income (%)** |  |  |  | 0.2 |
| ≤1.5x poverty level | 32 | 31.3 | 41.3 |  |
| >1.5x poverty level | 68 | 68.7 | 58.7 |  |
| **Employment (%)** |  |  |  | 0.2 |
| Wage-earning | 39.6 | 38.7 | 50 |  |
| Non-wage-earning | 60.4 | 61.3 | 50 |  |
| **BMI** | 32.9 (7.2) | 32.9 (7.2) | 32.8 (7.2) | 0.9 |
| **Smoking status (%)** |  |  |  |  |
| Former/current | 27.9 | 26.5 | 45.7 | 0.01 |
| **Alcohol use (%)** | 41.2 | 40.9 | 45.7 | 0.6 |
| **Nutrition (%)** |  |  |  | 0.7 |
| Poor | 49.6 | 49.7 | 47.8 |  |
| Intermediate | 49.4 | 49.2 | 52.2 |  |
| Ideal | 1 | 1.1 | 0 |  |
| **Physical Activity (%)** |  |  |  | 0.02 |
| Poor | 43.7 | 42.1 | 63 |  |
| Intermediate | 35.3 | 36.6 | 19.6 |  |
| Ideal | 21 | 21.3 | 17.4 |  |
| **Depression (%)** | 43.2 | 43.2 | 43.5 | 1 |
| **Diabetes status (%)** | 27.9 | 26.8 | 41.3 | 0.05 |
| **Hypertension status (%)** | 93.1 | 93.1 | 93.5 | 1 |
| **Antihypertensive medication use (%)** | 81.6 | 81 | 89.1 | 0.2 |
| **Chronic kidney disease (%)** | 20.7 | 19.2 | 39.1 | <0.01 |
| **Dyslipidemia (%)** | 62.7 | 62.2 | 69.6 | 0.4 |
| **Parental history of CVD (%)** | 53.7 | 53.5 | 56.5 | 0.8 |
|  |  |  |  |  |
| **Negative Moods** |  |  |  |  |
| **Anger in (median [IQR])** | 5 [3-7] | 5 [3-7] | 5 [2-7] | 0.8 |
| Mean (SD) | 5 (3.2) | 5 (3.2) | 4.9 (3) |  |
| **Anger out (median [IQR])** | 4 [2-6] | 4 [2-6] | 5 [3-6.8] | 0.09 |
| Mean (SD) | 4.1 (2.8) | 4 (2.8) | 4.8 (2.7) |  |
| **Hostility (median [IQR])** | 11 [8-14] | 11 [8-14] | 11 [8-14] | 0.6 |
| Mean (SD) | 11.1 (4.7) | 11.1 (4.6) | 11.5 (5) |  |
| **Religiosity** |  |  |  |  |
| **Spirituality (median [IQR])** | 24 [21-27] | 24 [21-27] | 24 [23.2-26] | 0.1 |
| Mean (SD) | 23.5 (4.8) | 23.4 (4.9) | 24.2 (3.2) |  |
| **Organized religion (median [IQR])** | 4 [4-5] | 4 [4-5] | 4 [4-5] | 0.4 |
| Mean (SD) | 4.1 (0.8) | 4.1 (0.8) | 4.2 (0.8) |  |
| **Non-organized religion (median [IQR])** | 7 [6-7] | 7 [6-7] | 7 [6-7] | 0.03 |
| Mean (SD) | 6.3 (1.2) | 6.3 (1.2) | 6.6 (0.7) |  |
| **Religious coping (median [IQR])** | 3 [2-3] | 3 [2-3] | 3 [2-3] | 0.6 |
| Mean (SD) | 2.6 (0.6) | 2.6 (0.6) | 2.6 (0.7) |  |
| **Discrimination** |  |  |  |  |
| **Daily discrimination (median [IQR])** | 7 [3-13] | 7 [3-13] | 8 [4.2-12] | 0.5 |
| Mean (SD) | 8.8 (8.4) | 8.8 (8.3) | 9.7 (8.6) |  |
| **Lifetime discrimination (median [IQR])** | 3 [2-4] | 3 [2-4] | 3 [1-3.8] | 0.04 |
| Mean (SD) | 3.1 (2) | 3.2 (2.1) | 2.6 (1.8) |  |
| **Burden of lifetime discrimination (median [IQR])** | 3.5 [2-5.5] | 3.5 [2-5.5] | 3.5 [1.5-4.5] | 0.4 |
| Mean (SD) | 3.7 (2.3) | 3.7 (2.3) | 3.4 (2.3) |  |
| **Negative Outlook** |  |  |  |  |
| **Pessimism (median [IQR])** | 2 [1-5] | 2 [1-5] | 3 [2-6] | 0.06 |
| Mean (SD) | 3.2 (2.9) | 3.1 (2.9) | 4 (2.8) |  |
| **John Henryism (median [IQR])** | 30 [27-33] | 30 [27-33] | 31 [27-33] | 0.9 |
| Mean (SD) | 29.7 (4.3) | 29.7 (4.3) | 29.6 (4.8) |  |
| **Negative coping resources** |  |  |  |  |
| **Social support (median [IQR])** | 6 [4-8] | 6 [4-8] | 5.5 [3-7] | 0.3 |
| Mean (SD) | 5.9 (2.7) | 5.9 (2.7) | 5.5 (2.7) |  |
| **Social status (median [IQR])** | 3 [2-5] | 3 [2-5] | 3 [2-5] | 0.6 |
| Mean (SD) | 3.4 (2) | 3.4 (2) | 3.6 (1.9) |  |
| **Stress (median [IQR])** | 4 [2-8] | 4 [2-8] | 3.5 [1.2-7] | 0.3 |
| Mean (SD) | 5 (4.1) | 5.1 (4.1) | 4.4 (3.8) |  |

**Appendix 6. Complete case analysis of the association between continuous and categorical component scores and risk of incident CVD.**

|  | **Per SD** |  | **Above/Below Median** |  | **Tertile of Component Score** | | |
| --- | --- | --- | --- | --- | --- | --- | --- |
| **No. of events/N: 46/609** |  |  |  |  |  |  |  |
| **Component Name** |  |  |  |  | **T1** | **T2** | **T3** |
| **Negative Moods** | 1.21 (0.89, 1.64) |  | 1.90 (0.99, 3.66) |  | 1 (REF) | 1.45 (0.65, 3.19) | 1.38 (0.61, 3.12) |
| **Religiosity** | 1.44 (1.02, 2.04) |  | 1.91 (1.01, 3.63) |  | 1 (REF) | 2.35 (1.05, 5.26) | 1.49 (0.63, 3.51) |
| **Discrimination** | 0.86 (0.63, 1.18) |  | 1.15 (0.62, 2.14) |  | 1 (REF) | 1.02 (0.50, 2.08) | 0.80 (0.37, 1.73) |
| **Negative Outlook** | 1.14 (0.84, 1.54) |  | 1.43 (0.77, 2.67) |  | 1 (REF) | 1.18 (0.54, 2.61) | 1.40 (0.66, 2.97) |
| **Negative coping resources** | 1.16 (0.84, 1.60) |  | 2.71 (1.43, 5.13) |  | 1 (REF) | 1.36 (0.64, 2.89) | 1.35 (1.00, 3.06) |

Models were adjusted for age, sex, diabetes status, dyslipidemia, hypertension, antihypertensive medication, chronic kidney disease, smoking status, body mass index, family history of CVD, education, income, and employment.

**Appendix 7. Complete case analysis of age and sex stratified association between SD increase in component score and risk of CVD.**

|  | **Age** | |  | **Sex** | |
| --- | --- | --- | --- | --- | --- |
| **No. of events/N: 46/609** | **< 57 years** | **≥ 57 years** |  | **Male** | **Female** |
|  | **15** | **31** |  | **18** | **28** |
| **Negative Moods** | 1.70 (0.89, 3.25) | 1.16 (0.80, 1.70) |  | 1.41 (0.77, 2.59) | 1.31 (0.88, 1.98) |
| **Religiosity** | 1.51 (0.81, 2.81) | 1.20 (0.75, 1.91) |  | 1.53 (0.86, 2.73) | 1.09 (0.62, 1.93) |
| **Discrimination** | 1.11 (0.59, 2.07) | 1.02 (0.69, 1.50) |  | 0.72 (0.39, 1.32) | 0.99 (0.65, 1.53) |
| **Negative Outlook** | 1.00 (0.47, 2.10) | 1.13 (0.78, 1.64) |  | 1.65 (0.91, 3.00) | 0.99 (0.65, 1.50) |
| **Negative coping resources** | 1.69 (0.88, 3.27) | 1.00 (0.63, 1.58) |  | 1.34 (0.73, 2.47) | 1.18 (0.74, 1.89) |

**Appendix 8a. Complete case analysis for association between continuous and categorical component and risk of incident CVD when including participants with missing CKD status.**

| **Cases/N**  **(69/862)** |  |  |  |  | **Tertile of Component Score** | | |
| --- | --- | --- | --- | --- | --- | --- | --- |
| **Component Name** | **Per SD** |  | **Above/Below Median** |  | **T1** | **T2** | **T3** |
| **Negative Moods** | 1.17 (0.90, 1.52) |  | 1.13 (0.68, 1.88) |  | 1 (REF) | 1.49 (0.79, 2.80) | 1.47 (0.76, 2.84) |
| **Religiosity** | 1.10 (0.84, 1.44) |  | 1.35 (0.81, 2.25) |  | 1 (REF) | 1.72 (0.91, 3.27) | 1.32 (0.66, 2.63) |
| **Discrimination** | 0.75 (0.58, 0.98) |  | 0.69 (0.41, 1.15) |  | 1 (REF) | 0.73 (0.41, 1.30) | 0.57 (0.30, 1.08) |
| **Negative Outlook*** | 1.17 (0.92, 1.50) |  | 1.23 (0.74, 2.06) |  | 1 (REF) | 1.10 (0.57, 2.12) | 1.31 (0.70, 2.45) |
| **Negative Coping Resources*** | 1.21 (0.94, 1.54) |  | 1.55 (0.95, 2.53) |  | 1 (REF) | 1.09 (0.58, 2.07) | 1.88 (1.04, 3.40) |

* In the main analysis, John Henryism loaded on Negative Outlook. In analysis that includes participants with missing CKD status, John Henryism loads on Negative Coping Resources.

**Appendix 8b.** **Complete case analysis for age and sex stratified association between SD increase in component and risk of incident CVD when including participants with missing CKD status.**

|  | **Age** | |  | **Sex** | |
| --- | --- | --- | --- | --- | --- |
| **Cases/N** | **< 58 years** | **≥ 58 years** |  | **Male** | **Female** |
| **69/862** | **23** | **46** |  | **26** | **43** |
| **Negative Moods** | 1.08 (0.70, 1.66) | 1.16 (0.84, 1.60) |  | 1.30 (0.83, 2.05) | 1.20 (0.85, 1.68) |
| **Religiosity** | 0.90 (0.62, 1.32) | 1.21 (0.81, 1.81) |  | 1.24 (0.81, 1.89) | 0.89 (0.59, 1.34) |
| **Discrimination** | 0.82 (0.50, 1.34) | 0.74 (0.53, 1.04) |  | 0.74 (0.46, 1.19) | 0.77 (0.53, 1.12) |
| **Negative Outlook*** | 1.13 (0.74, 1.72) | 1.15 (0.83, 1.59) |  | 1.16 (0.74, 1.82) | 1.11 (0.80, 1.55) |
| **Negative coping resources*** | 1.23 (0.83, 1.82) | 1.10 (0.79, 1.53) |  | 1.12 (0.73, 1.73) | 1.33 (0.96, 1.85) |

* In the main analysis, John Henryism loaded on Negative Outlook. In analysis that includes participants with missing CKD status, John Henryism loads on Negative Coping Resources.
